# Blood 1‐Deoxysphingolipid Levels Are Associated With Epidermal Denervation in Small Fiber Neuropathy

**DOI:** 10.1111/jns.70089

**Published:** 2025-12-11

**Authors:** Luisa Kreß, Caren Meyer zu Altenschildesche, Nadine Egenolf, Claudia Sommer, Thorsten Hornemann, Nurcan Üçeyler

**Affiliations:** ^1^ Department of Neurology University Hospital Würzburg Würzburg Germany; ^2^ Department of Biochemistry University of Zurich Zurich Switzerland

**Keywords:** epidermal denervation, lipoproteins, small fiber neuropathy, sphingolipids

## Abstract

**Background and Aims:**

Dysfunctional sphingolipid metabolism leads to nerve fiber degeneration, particularly of small caliber Aδ and C fibers, in hereditary sensory and autonomic neuropathies. We aimed to investigate the association of blood 1‐deoxysphingolipid (1‐deoxySL) profiles and skin denervation in idiopathic small fiber neuropathy (SFN).

**Methods:**

Seventy‐five patients with idiopathic SFN were recruited prospectively. Patients underwent a skin punch biopsy at the lower leg and upper thigh for intraepidermal nerve fiber density (IENFD) quantification. IENFD was correlated with individual blood 1‐deoxySL levels, amino acid profiles, and determinants of glucose and lipoprotein metabolism.

**Results:**

Median distal IENFD in SFN patients was 5.3 fibers/mm. In 38/75 (51%) patients, IENFD was ≤ 5.3 fibers/mm (i.e., “low fiber density,” LFD), while in 37/75 (49%) patients, IENFD was > 5.3 fibers/mm (i.e., “high fiber density,” HFD). 1‐deoxySL was higher in patients with LFD compared to HFD (*p* < 0.05). Fasting plasma glucose was also higher in patients with LFD than in patients with HFD (*p* < 0.01), while HDL was lower in patients with LFD compared to HFD (*p* < 0.001).

**Interpretation:**

1‐deoxySL, lipoprotein metabolism, and glucose levels are associated with the extent of epidermal denervation in SFN, supporting the role of this metabolic axis in small nerve fiber pathology.

## 1 Introduction

Small fiber neuropathy (SFN) is characterized by damage to the small‐diameter Aδ and C nerve fibers, leading to neuropathic pain and altered thermal perception, which may impair patients' health‐related quality of life [1]. In more than 50% of cases, the etiology remains idiopathic even after thorough diagnostic work‐up. The SFN diagnosis involves clinical evaluation and specialized small fiber tests, with skin punch biopsy widely recognized as a key diagnostic tool [2, 3, 4]. The typical finding in SFN is the reduction of skin innervation [5, 6, 7]. The mechanisms underlying epidermal denervation are diverse [6] and recent studies suggest that alterations in sphingolipid (SL) metabolism may play a role in driving these structural changes [8]. SL are important components of neuronal membranes and can be metabolized into complex SL with potential neurotoxic effects [9, 10].

SL contributes to peripheral neuropathies by modulating inflammation, apoptosis, and axonal degeneration [11]. Serine palmitoyltransferase (SPT) catalyzes the first and rate‐limiting step in SL de novo synthesis. Located in the endoplasmic reticulum, SPT typically conjugates serine (ser) and palmitoyl‐CoA. To a minor extent, SPT can also metabolize alanine (ala) and glycine as alternative substrates, producing an atypical class of 1‐deoxysphingolipid (1‐deoxySL) [12]. 1‐deoxySL accumulates as metabolic intermediates due to their resistance to canonical SL degradation and are instead processed via a cytochrome P450‐dependent pathway [13]. Pathologically, 1‐deoxySL drives hereditary sensory and autonomic neuropathy type 1 (HSAN1), where SPT mutations cause a persistent shift in substrate specificity from ser to ala [14]. HSAN1 is a rare monogenic axonopathy characterized by multimodal sensory loss, neuropathic pain, anhidrosis, and ulcers [15, 16]. Elevated 1‐deoxySL disrupts mitochondrial function, cytoskeletal reorganization, and axon formation [10, 13, 17]. 1‐deoxySL is also elevated in type 2 diabetes mellitus (DM), linked to small fiber dysfunction [8, 18]. In mice, l‐ser restriction combined with obesity, insulin resistance, and dyslipidemia enhances 1‐deoxySL formation and impairs small nerve fibers [19].

We investigated the interplay between 1‐deoxySL formation, glucose, and amino acid (AA) metabolism as potential contributors to skin denervation in idiopathic SFN.

## 2 Methods

### 2.1 Patients

Between May 2015 and December 2019, we prospectively recruited adult patients with a medical history and clinical phenotype indicative of SFN at the Department of Neurology, University Hospital Würzburg, Germany as part of a larger study summarizing the results of in‐depth clinical characterization, small fiber testing, and sequencing of pain‐associated genes [2]. Patients with large fiber involvement on clinical examination and/or nerve conduction studies, as well as those with potential causes of SFN (e.g., prior diagnosis of DM, renal insufficiency, untreated thyroid dysfunction, vitamin B12 deficiency, or acute systemic infection) were excluded. Individuals with severe psychiatric disorders, recent malignancy (past 5 years), substance abuse, or habitual alcohol consumption were also ineligible. All participants gave written informed consent before inclusion, and our study was approved by the Ethics Committee of the University of Würzburg Medical Faculty (#135/15).

### 2.2 Clinical Assessment and Nerve Conduction Studies

All patients underwent a medical history interview focused on pain, a complete neurological examination, and pain assessment using the numeric rating scale (NRS, 0 = no pain to 10 = worst pain). Patients filled in the German versions of the Neuropathic Pain Symptom Inventory (NPSI) [20, 21] and the Graded Chronic Pain Scale (GCPS) [22]. To exclude large fiber polyneuropathy, nerve conduction studies of the sural and tibial nerves were performed following established protocols and compared with laboratory normative values.

### 2.3 Laboratory Tests

Venous blood was drawn for serum and plasma samples between 8 and 9 am after overnight fasting, no heavy meals and alcohol consumption, or intensive physical activity the day before. Plasma SL and AA profiles were analyzed at the Institute of Biochemistry, University of Zurich, Switzerland. As SL are conjugated to different O‐linked head groups and *N*‐acyl chains in plasma, an acid/base hydrolysis was done before analysis to quantify the total SL base profile in plasma [23]. Thus, the reported SL base concentrations reflect the total SL base compositions, free and *N*‐acetylated. SL analysis was performed as previously described [23] and included the following targets: C16‐sphingosine, C16‐sphinganine, C17‐sphingosine, C17‐sphinganine, phyto‐C18‐sphingosine, C18SAdiene, C18‐sphingosine, C18‐sphinganine, totalSL, C19‐sphingosine, C19‐sphinganine, anteiso‐branched‐C18‐sphingosine, anteiso‐branched‐C18‐sphinganine, C20‐sphingosine, C20‐sphinganine, 1‐deoxysphingosine (14Z) (1‐deoxySO), 1‐deoxysphinganine (1‐deoxySA), total SL, deoxymethylsphingosine, and deoxymethylsphinganine. Briefly, 100 μL plasma was supplemented with 0.5 mL methanol (200 pmol d7‐sphingosine and d7‐sphinganine (Avanti Polar Lipids, Alabaster, AL, USA) included). Samples were incubated on a thermo‐mixer (1 h, 37°C) to precipitate proteins. After centrifugation (16 000*g*, 5 min, room temperature [RT]), transferring the supernatant to a new tube, and adding 75 μL methanolic HCl (1 N HCl and 10 M H_2_O in methanol), suspension was incubated (16 h, 65°C). To neutralize HCl, 100 μL of 10 M KOH was added. This step was followed by adding 625 μL chloroform, 100 μL 2 N ammonium hydroxide, and 0.5 mL alkaline water. After vortexing, centrifugation (16 000*g*, 5 min, RT), and discarding the upper phase, the lower phase was washed 2–3 times with alkaline water (pH 10.3). Samples were dried under N2 and separated on C18 column (Uptispere 120 Å, 5 μm, 125 × 2 mm, Interchim, Montluçon, France). Analysis was done using a TSQ Quantum Ultra mass spectrometer (Thermo Fisher Scientific, Waltham, MA, USA).

The following plasma AA levels were determined: ala, ser, ala/ser ratio, arginine, aspartic acid, cystine, glutamic acid, glycine, histidine, isoleucine, leucine, lysine, methionine, phenylalanine, proline, threonine, tyrosine, and valine. AA were analyzed in 10 μL plasma as described before [24]. Briefly, 180 μL ice‐cold methanol (1 nmol stable isotope–labeled AA [Cambridge Isotope Laboratories, MSK‐A2‐1.2] included) was added to precipitate plasma. After incubation (30 min, −20°C), centrifugation (14 000 *g*, 10 min, 4°C), and transferring the supernatant to a fresh tube, pellets were dried under N_2_ stream. Pellets were resuspended in 100 μL of 0.1% acetic acid, shifted to an autosampler, and centrifuged (16 000*g*, 5 min). A reverse‐phase C18 column (EC 250/2 NUCLEOSIL 100‐3 C18HD, length 250 mm, internal diameter 2 mm; Macherey‐Nagel) was used to separate AA. For sample analysis, liquid chromatography and multiple reaction monitoring mass spectrometry (QTRAP 6500 + LC‐MS/MS‐MS System, SCIEX, Darmstadt, Germany) were applied. Details on the procedure were already published [24]. MultiQuant 2.1 software (SCIEX, Darmstadt, Germany) was used for quantification.

Further, we determined fasting plasma glucose levels, measured hemoglobin A1c (HbA1c), and performed an oral glucose tolerance test (OGTT). Impaired glucose tolerance was defined as a 2‐h glucose level ≥ 140 mg/dL and < 200 mg/dL upon intake of 75 g glucose/300 mL. Patients diagnosed with DM based on the criteria of the American Diabetes Association [25] were excluded from the study. We measured serum high‐ and low‐density lipoproteins (HDL, LDL), triglycerides (TG), and cholesterol levels. Non‐HDL was calculated by subtracting HDL cholesterol from total cholesterol.

### 2.4 Skin Punch Biopsy

Six‐millimeter skin punch biopsies were obtained from the right lateral distal calf and proximal thigh of all patients following a standard procedure [26]. IENFD was determined using PGP9.5 immunofluorescence staining [26] and quantified according to established counting rules [27]. Results were compared with the normative values established in our laboratory, using the same staining and quantification protocols. These laboratory reference values were derived from a cohort of 180 healthy controls (124 women; median age: 50 years, 20–84; 56 men; median age: 53 years, 22–76) based on skin biopsies obtained from the lower leg (women: *n* = 109) and the upper thigh (women: *n* = 102). IENFD was considered abnormal when < 6 fibers/mm were found at the distal site or < 8 fibers/mm at the proximal site.

### 2.5 Statistical Analysis

Statistical analysis was performed using SPSS Statistics 29 (IBM, Ehningen, Germany). A priori power calculation was not performed for the subgroup analysis, as the study was exploratory in nature and was based on available patient data. To test for normal distribution, the Shapiro–Wilk test was used. Normally distributed data were analyzed applying the *t* test; non‐normally distributed data were assessed using the non‐parametric Mann–Whitney *U* test. In case of unequal group sizes, the exact non‐parametric Mann–Whitney *U* test was applied, and effect sizes were expressed as Hodges–Lehmann median differences with 95% confidence intervals. For categorical data, Fisher's exact test and the *χ* ^2^ test were used. Spearman's correlation coefficient was calculated. GraphPad Prism (9.5.1, San Diego, CA, USA) software was used to create violin, box, and scatter plots as well as correlation analysis. *p* < 0.05 was assumed statistically significant. To further explore the correlation between demographic factors (age, sex) or metabolic parameters and IENFD, we additionally performed a multiple linear regression analysis and treated IENFD as a continuous outcome variable. Given the substantial multicollinearity among predictors (condition index > 30, variance inflation factor > 5), a stepwise multiple regression approach was subsequently performed to identify independent predictors while minimizing collinearity effects. Variables were entered according to statistical significance (entry criterion *p* < 0.05, removal criterion *p* > 0.10). To quantify each predictor's independent contribution to IENFD, both unstandardized (*B*) and standardized (*β*) regression coefficients were reported. Adjusted *R* ^2^ was used to assess the goodness of fit while accounting for the number of predictors included in the model.

## 3 Results

### 3.1 Clinical Characteristics

**FIGURE 1:**
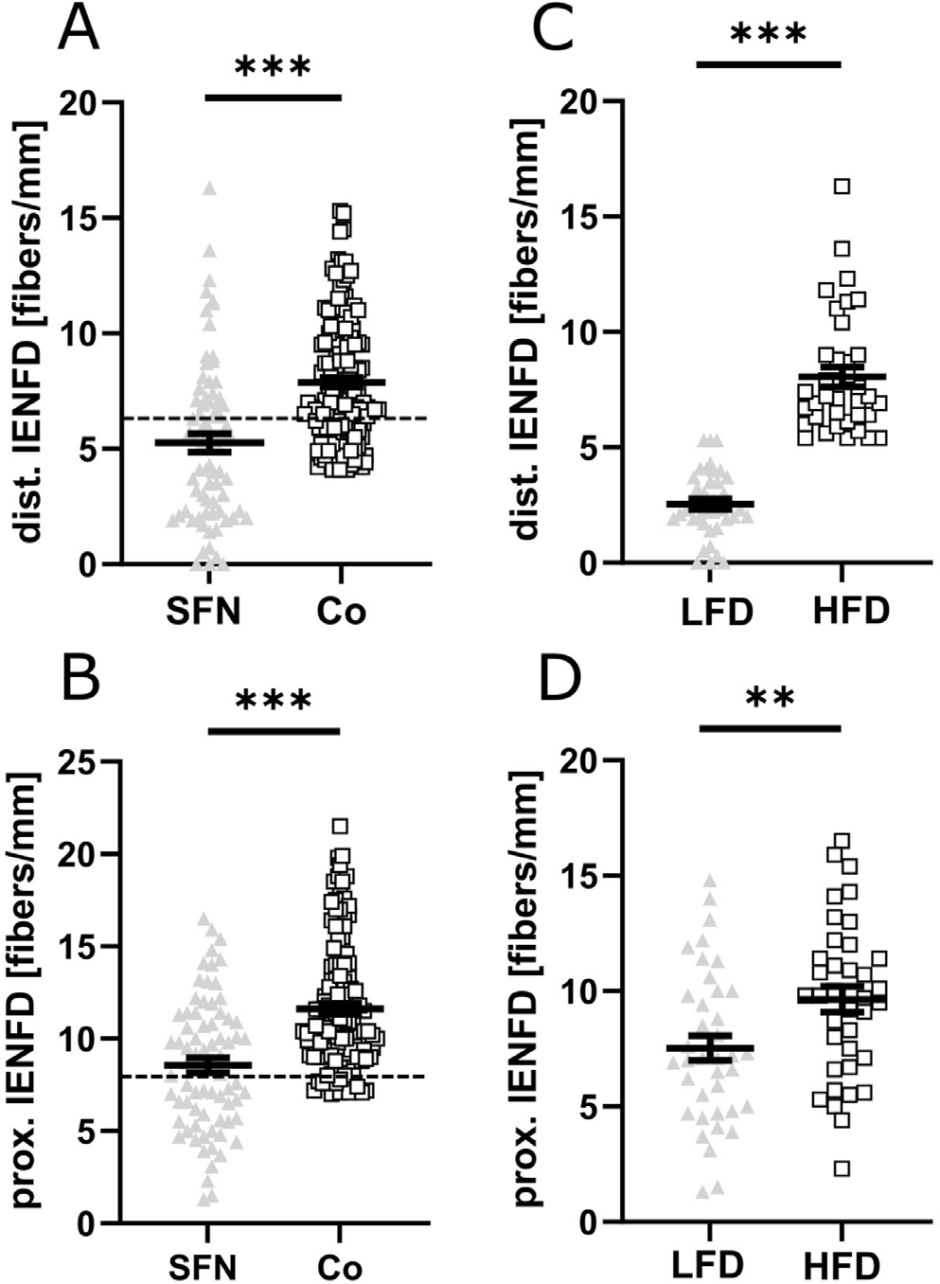
Skin punch biopsy results. The scatter plots show the IENFD in fibers/mm on skin punch biopsies from SFN patients and Co at the distal (A) and proximal (B) biopsy sites. The dashed line indicates the diagnostic threshold for pathological IENFD based on our laboratory's normative data. IENFD was reduced in SFN patients compared to healthy controls at the distal and proximal sites (*p* < 0.001 each), indicating small fiber loss as a hallmark of SFN. Scatter plots (C and D) depict a post hoc subgroup analysis of SFN patients stratified by the median IENFD into LFD and HFD subgroups for distal (C) and proximal (D) sites. Reduced IENFD in the LFD subgroup reflects more severe epidermal denervation. Number of samples investigated: SFN = 75 Co = 180, LFD = 38, HFD = 37. Co = healthy controls, dist. = distal, HFD = high fiber density, IENFD = intraepidermal nerve fiber density, LFD = low fiber density, prox. = proximal. **p < 0.01; ***p < 0.001.

Clinical characteristics of the study population are summarized in Table 1. We recruited 75 patients (43 women, median age 52 years, range 19–78) who reported neuropathic pain with a median current pain intensity of 4/10 NRS. Median IENFD was reduced compared to control values (lower leg 5.3 fibers/mm, range 0–16.3, *p* < 0.001, Figure 1A) and was normal at the upper thigh (8.5 fibers/mm, range 1.3–16.5, Figure 1B). Based on the overall median of the 75 patients, the cohort was divided into a “low fiber density” (LFD) (median 2.3 fibers/mm, 0–5.3 fibers/mm; *n* = 38) and “high fiber density” (HFD) (median 7.2 fibers/mm, 5.4–16.3 fibers/mm; *n* = 37) group (Figure 1C,D; Table 1). Biometric data, pain phenotype, neurological parameters, and symptoms of metabolic syndrome were not different between the two groups, except for a higher body mass index (BMI) in LFD compared to HFD (*p* < 0.01, Table 1).

**TABLE 1:** Clinical characteristics of the study cohort.

	Small fiber neuropathy (*n* = 75)	LFD (*n* = 38)	HFD (*n* = 37)	*p* LFD vs HFD
F/M	43/32	17/21	27/10	*p* < 0.05
Median age (years)	52 (19–78)	53.5 (31–78)	49 (19–73)	n.s.
Median current pain intensity GCPS on NRS	4 (0–8)	4 (0–8)	4 (1–8)	n.s.
Median NPSI sum score	31 (0–85)	31 (0–85)	34 (7–69)	n.s.
Genetic variant	13	7	6	n.s.
Parameters related to metabolism				
BMI (kg/m^2^)	25.6 (17.5–42.2)	26 (20.2–42.2)	24.9 (17.5–37.6)	*p* < 0.05
Arterial hypertension	10/75 (13%)^d^	7/38 (18%)	3/37 (8%)	n.s.
Dyslipidemia	4/75 (5%)	2/38 (5%)	2/37 (5%)	n.s.
Hypercholes‐terolemia	5/75 (7%)^e^	4/38 (11%)	1/37 (3%)	n.s.
Neurological examination^c^				
Negative sensory signs^a^	29/75 (39%)	17/38 (45%)	12/37 (32%)	n.s.
Positive sensory signs^b^	18/75 (24%)	8/38 (21%)	10/370 (27%)	n.s.
Nerve conduction studies				
Sural nerve				
SNAP (μV)	17.0 (3.4–41.7)	13.8 (3.4–28.0)	19.0 (4.1–41.7)	*p* < 0.01
NCV (m/s)	47.1 (38.7–58.0)	47.5 (38.7–54.2)	47.0 (39.2–58.0)	n.s.
Tibial nerve				
CMAP, distal stimulation (mV)	19.3 (2.1–35.3)	18.9 (2.1–35.3)	19.7 (10.0–34.3)	n.s.
CMAP, proximal stimulation (mV)	14.8 (5–28.5)	13.73 (5–28.0)	15.8 (8.9–28.5)	n.s.
dmL	3.7 (2.8–5.6)	3.7 (2.8–5.6)	3.8 (2.8–5.1)	n.s.
NCV (m/s)	46.1 (39.5–67.0)	45.7 (39.5–67.0)	47.7 (39.8–54.2)	n.s.
IENFD distal (fibers/mm)	5.3 (0–16.3)	2.3 (0–5.3)	7.2 (5.4–16.3)	*p* < 0.001
IENFD proximal (fibers/mm)	8.5 (1.3–16.5)	7.1 (1.3–14.8)	9.8 (2.3–16.5)	*p* < 0.01
LDL (mM)	3.3 (1.2–5.0)	3.3 (1.2–5.0)	3.3 (2.0–4.7)	n.s.
HDL (mM)	1.4 (0.6–2.9)	1.2 (0.6–2.7)	1.6 (0.8–2.9)	*p* < 0.001
Non‐HDL (mM)	3.9 (2.0–6.5)	4.2 (2.0–5.6)	3.8 (2.3–6.5)	n.s.
Cholesterol (mM)	5.4 (3.2–7.3)	5.4 (3.2–7.0)	5.6 (4.0–7.3)	n.s.
TG (mM)	1.2 (0.5–5.1)	1.4 (0.5–5.1)	1.2 (0.5–4.2)	n.s.
OGTT, fasting (mg/dL)	98.0 (74.0–118.0)	102.0 (81.0–118.0)	94.0 (74.0–113.0)	*p* < 0.01
OGTT, 2 h (mg/dL)	119.0 (66.0–186.0)	121.0 (66.0–186.0)	119.0 (79.0–170.0)	n.s.
HbA1c (%)	5.5 (3.6–6.2)	5.5 (3.6–6.2)	5.4 (4.5–5.9)	n.s.

Abbreviations: BMI = body mass index, CMAP = compound muscle action potential, dmL = distal motor latency, F = female, GCPS = graded chronic pain scale, HbA1c = hemoglobin A1c, HDL = high density lipoprotein, HFD = high fiber density, IENFD = intraepidermal nerve fiber density, LDL = low density lipoprotein, LFD = low fiber density, M = male, NCV = nerve conduction velocity, Non‐HDL = non‐high density lipoprotein, NPSI = neuropathic pain symptom inventory, n.s. = not significant, NRS = numeric rating scale, OGTT = oral glucose tolerance test, SNAP = sensory nerve action potential, TG = triglycerides.

^a^ Hypoesthesia, thermal hypoesthesia, hypoalgesia.

^b^ Hyperalgesia, allodynia.

^c^ Seven patients with pathological results in ENG compared to laboratory internal normative values. Since these patients showed no other signs of large fiber neuropathy and a single parameter was abnormal in the electrophysiological measurements, they were not excluded.

^d^ All patients on antihypertensive treatment.

^e^ Two patients on statins. Data are given as median and range in brackets.

### 3.2 High Levels of 1‐deoxySL Correlated With Low IENFD

**FIGURE 2:**
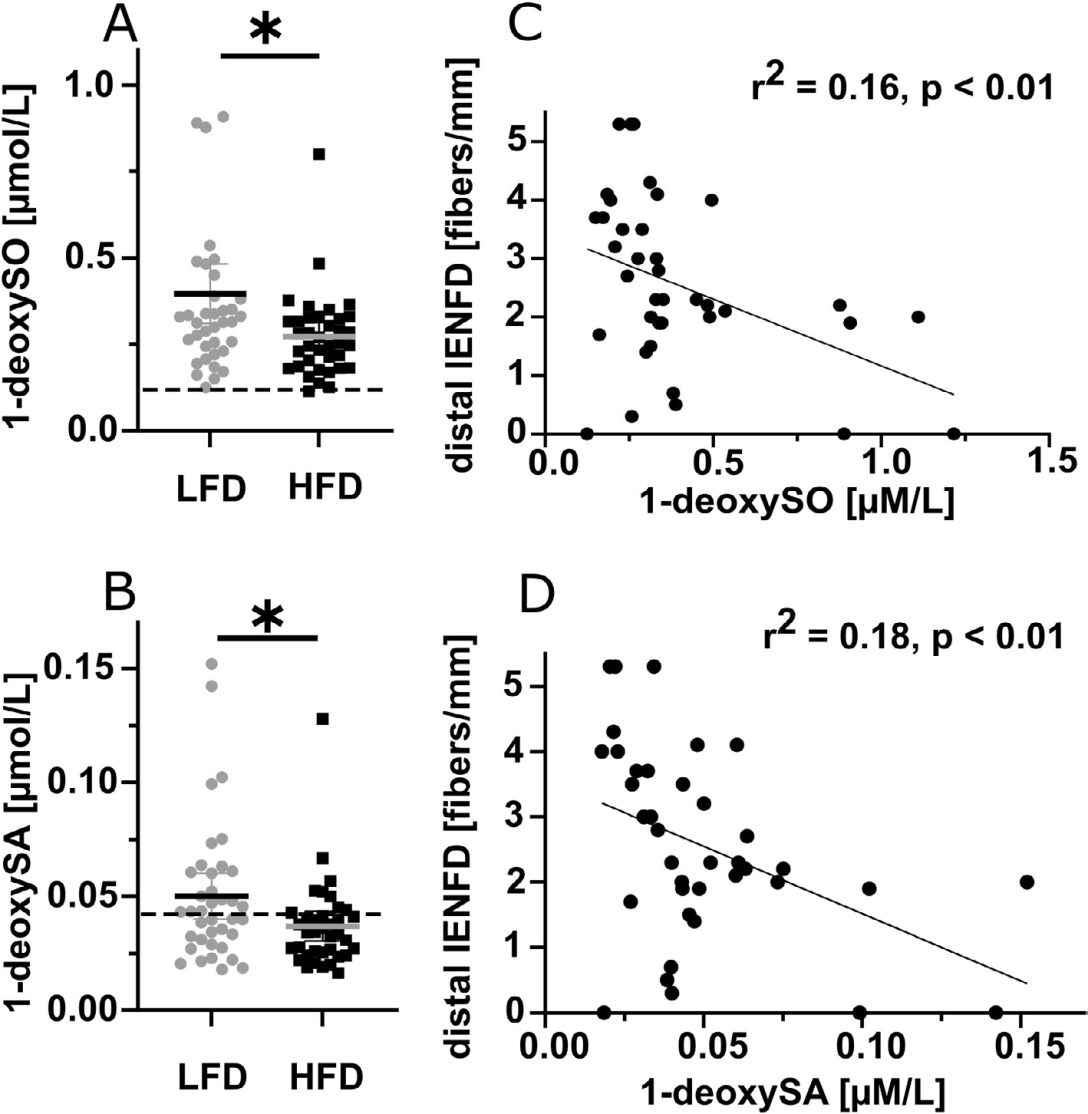
Plasma 1‐deoxySL levels and IENFD. The box plots (A and B) show the plasma levels of (A) 1‐deoxySO and (B) 1‐deoxySA. Levels of 1‐deoxySO and 1‐deoxySA were higher in SFN patients within the LFD subgroup compared to those within the HFD subgroup. These findings support a link between elevated 1‐deoxySL levels and more severe nerve fiber loss. The scatter plots (C and D) show the negative correlation of (C) 1‐deoxySO and (D) 1‐deoxySA with IENFD in patients with LFD while not in patients with HFD. Number of samples investigated: LFD = 38, HFD = 37. 1‐deoxySA = 1‐deoxy‐sphinganine, 1‐deoxySO = 1‐deoxy‐sphingosine (14Z), dist. = distal, HFD = high fiber density, IENFD = intraepidermal nerve fiber density, LFD = low fiber density. *p < 0.05.

1‐deoxySO, 1‐deoxySA, as well as total 1‐deoxySL were higher in the LFD compared to the HFD group (*p* < 0.05 each, Figure 2A,B, Table 2). In the LFD group, we observed an inverse correlation between IENFD and 1‐deoxySO (*r* ^2^ = 0.16, *p* < 0.01) and 1‐deoxySA (*r* ^2^ = 0.18, *p* < 0.01) (Figure 2C,D). Other SL profiles remained without difference in intergroup comparison (Table 2).

**TABLE 2:** SL and amino acids of study cohort.

	Small fiber neuropathy (*n* = 75)	LFD (*n* = 38)	HFD (*n* = 37)	*p* LFD vs. HFD
C16‐sphingosine (μmol/L)	8.93 (4.44–31.80)	8.17 (4.44–31.62)	9.80 (5.46–31.80)	n.s.
C16‐sphinganine (μmol/L)	0.31 (0.11–0.84)	0.30 (0.11–0.78)	0.33 (0.13–0.84)	n.s.
C17‐sphingosine (μmol/L)	5.79 (2.66–16.24)	5.52 (2.66–14.27)	6.86 (3.53–16.25)	*p* < 0.01
C17‐sphinganine (μmol/L)	0.08 (0.03–0.19)	0.08 (0.03–0.17)	0.09 (0.04–0.19)	n.s.
Phyto‐C18‐sphingosine (μmol/L)	0.88 (0.31–2.13)	0.85 (0.31–1.84)	0.91 (0.31–2.13)	n.s.
C18‐SAdiene (μmol/L)	17.57 (6.82–39.27)	16.36 (9.43–36.87)	18.40 (6.82–39.27)	*p* < 0.05
C18‐sphingosine (μmol/L)	62.92 (43.00–94.29)	62.24 (46.10–84.24)	63.05 (43.00–94.29)	n.s.
C18‐sphinganine (μmol/L)	2.71 (1.19–4.48)	2.74 (1.19–4.48)	2.61 (1.49–4.01)	n.s.
Sphingolipids, total (μmol/L)	65.37 (45.86–97.38)	64.72 (47.72–87.69)	66.39 (45.81–97.38)	n.s.
C19‐sphingosine (μmol/L)	0.13 (0.01–0.22)	0.13 (0.08–0.20)	0.14 (0.01–0.22)	n.s.
C19‐sphinganine (μmol/L)	0.01 (< 0.01 to 0.03)	0.01 (< 0.01 to 0.03)	0.01 (< 0.01 to 0.02)	n.s.
Anteiso‐branched C18‐sphingosine (meC18SO) (μmol/L)	2.86 (0.02–12.82)	2.59 (0.02–12.82)	3.21 (0.08–7.67)	n.s.
Anteiso‐branched C18‐sphinganine (meC18SA) (μmol/L)	0.03 (0.02–0.38)	0.03 (0.02–0.20)	0.03 (0.02–0.38)	n.s.
C20‐Sphingosine (μmol/L)	0.26 (0.04–0.38)	0.26 (0.09–0.45)	0.25 (0.06–0.41)	n.s.
C20‐sphinganine (μmol/L)	0.03 (< 0.01–0.10)	0.03 (0.01–0.10)	0.03 (< 0.01–0.06)	n.s.
1‐Deoxysphingosine (14Z) (μmol/L)	0.29 (0.11–1.22)	0.33 (0.01–0.10)	0.25 (0.11–0.80)	*p* < 0.05
1‐Deoxysphinganine (μmol/L)	0.04 (0.02–0.15)	0.04 (0.02–0.15)	0.03 (0.02–0.13)	*p* < 0.05
1‐Deoxysphingolipids, total (μmol/L)	0.33 (0.13–1.36)	0.36 (0.14–1.36)	0.29 (0.13–0.93)	*p* < 0.05
Deoxymethylsphingosine (μmol/L)	< 0.01 (< 0.01 to > 0.01)	< 0.01 (< 0.01 to < 0.01)	< 0.01 (< 0.01 to < 0.01)	n.s.
Deoxymethylsphinganine (μmol/L)	< 0.01 (< 0.0 to < 0.01)	< 0.01 (> 0.01 to < 0.01)	< 0.01 (< 0.01 to < 0.01)	n.s.
Alanine (mM)	246.90 (159.51–520.75)	241.29 (162.91–520.75)	248.60 (159.51–452.36)	n.s.
Serine (mM)	57.05 (26.70–95.68)	54.93 (26.70–82.67)	57.31 (32.48–95.68)	n.s.
Alanine/serine (ratio)	4.29 (2.71–9.78)	4.40 (3.05–9.78)	4.20 (2.71–6.15)	n.s.
Arginine (mM)	48.68 (23.36–111.61)	47.44 (23.36–92.69)	50.43 (24.80–111.61)	n.s.
Aspartic acid (mM)	86.46 (51.83–147.42)	86.94 (59.24–147.42)	85.89 (51.83–131.78)	n.s.
Cystine (mM)	1.06 (0.14–5.86)	0.97 (0.22–5.86)	1.2 (0.14–5.54)	n.s.
Glutamic acid (mM)	222.22 (43.33–595.75)	256.95 (101.77–407.85)	194.60 (43.33–595.75)	*p* < 0.05
Glycine (mM)	156.70 (71.10–321.96)	143.92 (93.59–252.32)	173.89 (71.10–321.96)	n.s.
Histidine (mM)	86.07 (60.68–129.88)	85.66 (64.80–129.88)	87.12 (60.68–126.55)	n.s.
Isoleucine (mM)	118.35 (73.39–199.44)	116.72 (73.39–199.44)	119.13 (75.90–182.51)	n.s.
Leucine (mM)	49.08 (28.71–89.11)	49.16 (28.71–78.23)	467.29 (33.86–89.11)	n.s.
Lysine (mM)	168.08 (104.30–295.51)	169.25 (111.51–254.50)	167.62 (104.30–295.51)	n.s.
Methionine (mM)	8.05 (3.40–31.99)	7.95 (3.40–31.99)	8.43 (3.60–31.96)	n.s.
Phenylalanine (mM)	61.53 (43.47–99.72)	59.77 (43.47–99.72)	62.30 (46.54–85.26)	n.s.
Proline (mM)	222.58 (107.29–355.30)	241.02 (120.34–355.30)	252.04 (165.35–369.48)	n.s.
Threonine (mM)	126.33 (78.43–210.24)	126.92 (78.43–210.24)	126.6 (85.20–197.66)	n.s.
Tyrosine (mM)	53.71 (35.84–94.13)	53.32 (35.84–94.13)	53.62 (36.74–89.66)	n.s.
Valine (mM)	252.04 (165.35–421.70)	254.77 (168.05–421.70)	251.30 (165.35–369.48)	n.s.

*Note:* Data are given as median and range in brackets.

Abbreviations: HFD = high fiber density, LFD = low fiber density, SL = sphingolipids.

### 3.3 1‐DeoxySL Levels Correlate With Sensory Profiles Only in Patients With LFD

We have further examined correlations between plasma concentrations of 1‐deoxySO, 1‐deoxySA, total 1‐deoxySL, and quantitative sensory testing (QST) parameters. For this, z‐transformed QST values from our previously published cohort [2] were used. In the LFD group, 1‐deoxySO and total 1‐deoxySL showed a weak positive correlation with the cold pain threshold (*r* ^2^ = 0.31, *p* < 0.01 and *r* ^2^ = 0.30, *p* < 0.05, Table S1). All SL parameters positively correlated with paradoxical heat sensation (*r* ^2^ between 0.20 and 0.26, *p* < 0.05, each, Table S1). In the HFD group, no correlations were observed between 1‐deoxySL levels and QST sensory profiles (Table S1).

### 3.4 Ala/ser Ratio Correlates With 1‐DeoxySA Only in Patients With LFD

**FIGURE 3:**
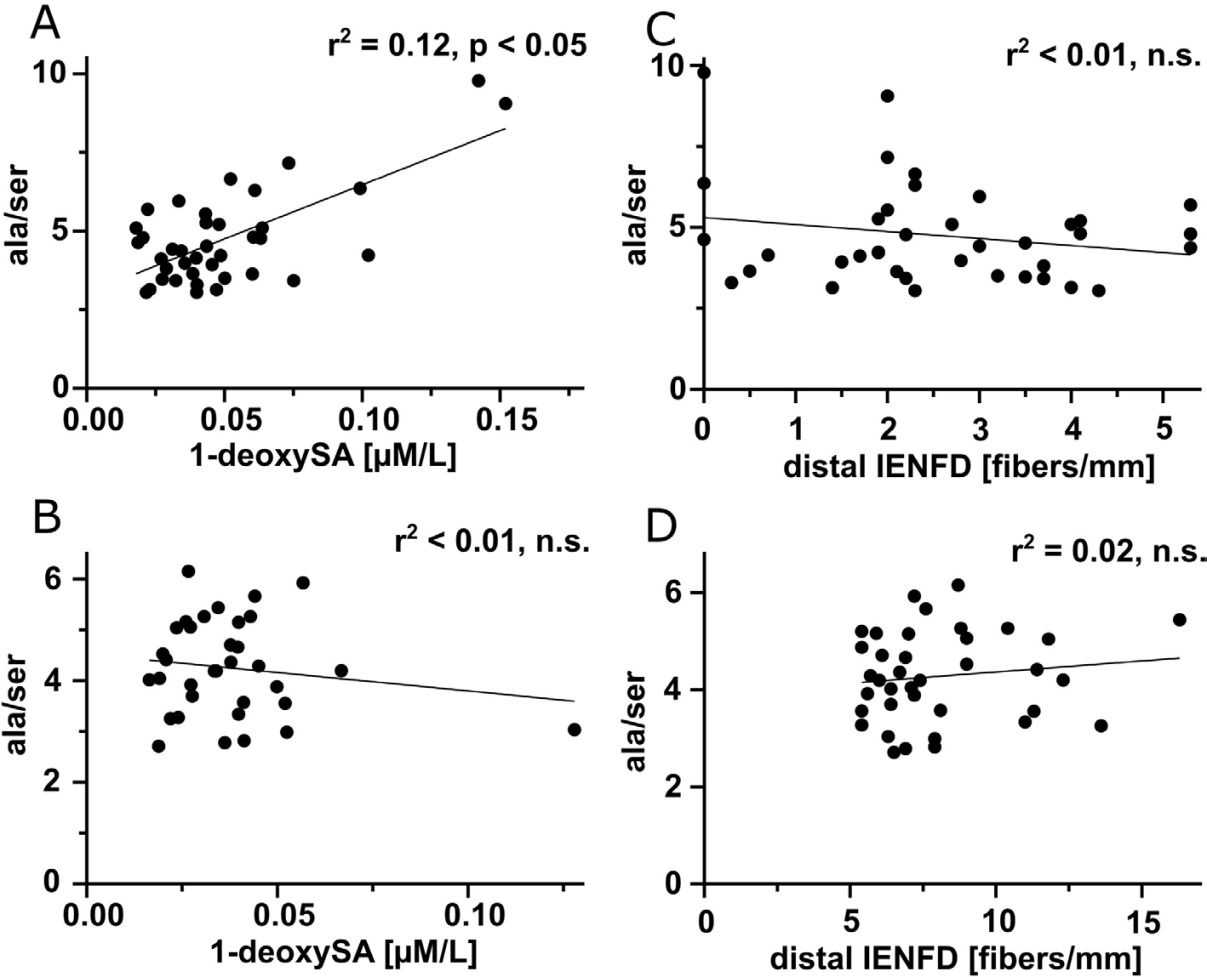
Ala/ser ratio in LFD and HFD. The scatter plots (A–D) show the correlation of the ala/ser ratio and 1‐deoxySA in LFD (A), 1‐deoxySA in HFD (B), IENFD in LFD (C), and IENFD in HFD (D). In LFD, the ala/ser ratio correlates with 1‐deoxySA levels (*p* < 0.05). Number of samples investigated: LFD = 38, HFD = 37. 1‐deoxySA = 1‐deoxy‐sphinganine, ala/ser = alanine/serine, HFD = high fiber density, IENFD = intraepidermal nerve fiber density, LFD = low fiber density, ns = not significant.

**FIGURE 4:**
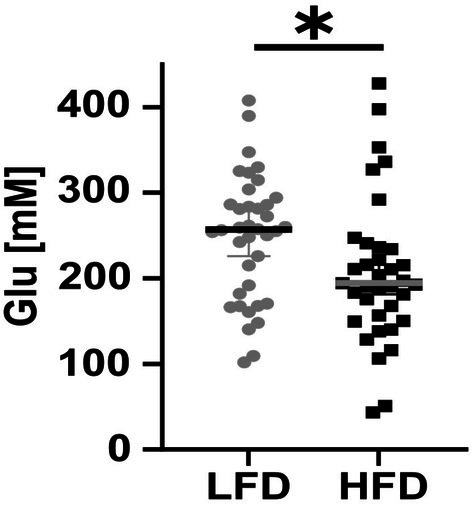
Glutamic acid in LFD and HFD. The box plots show that the total levels of glutamic acid are higher in patients with LFD than in patients with HFD. Number of samples investigated: LFD = 38, HFD = 37. Glu = glutamic acid, HFD = high fiber density, LFD = low fiber density. *p < 0.05.

Total l‐ala and l‐ser as well as the ala/ser ratio showed no difference between patients with LFD and those with HFD (Figure S1). In the LFD group, we observed a correlation between 1‐deoxySL levels and the ala/ser ratio (Figure 3A), which was not present in the HFD group (Figure 3B). In both groups, no association was found between IENFD and the ala/ser ratio (Figure 3C,D). Among the other AA, glutamic acid was higher in patients with LFD compared to patients with HFD (*p* < 0.05, Figure 4, Table 2). Other AA did not differ between groups (Figure S2, Table 2).

### 3.5 High Levels of Glucose and Lipids Are Associated With Lower Epidermal Innervation

**FIGURE 5:**
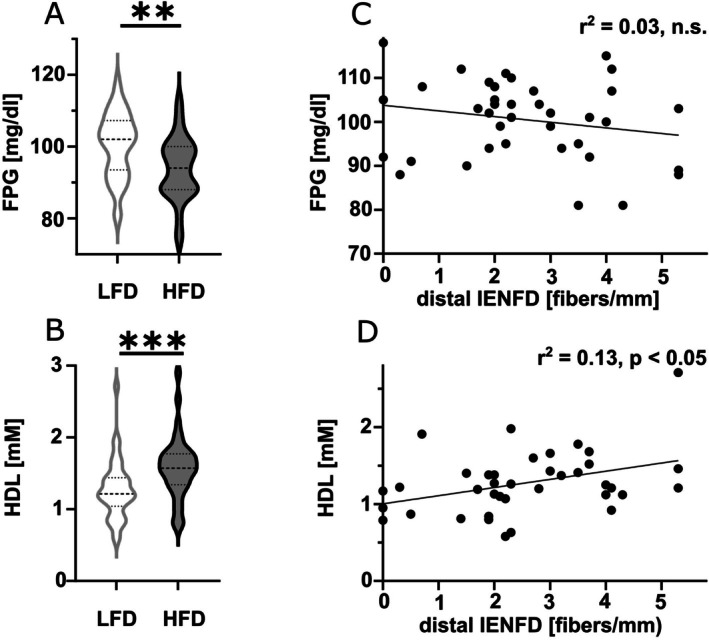
Metabolic markers and skin innervation. The violin plots show (A) a higher FPG and (B) lower HDL levels in patients with LFD compared to patients with HFD. The scatter plots (C) show no correlation between fasting plasma glucose and IENFD in patients with LFD, and (D) a positive correlation between HDL and IENFD in patients with LFD. Number of samples investigated: LFD = 38, HFD = 37. FPG = fasting plasma glucose, HDL = high density lipoprotein, HFD = high fiber density, IENFD = intraepidermal nerve fiber density, LFD = low fiber density, TG = triglycerides. **p < 0.01; ***p < 0.001.

To investigate potential metabolic links, we analyzed fasting glucose, HbA1c, TG, and cholesterol levels (Table 1). Fasting plasma glucose was higher (*p* < 0.01) and HDL lower (*p* < 0.001) in the LFD group compared to the HFD group (Figure 5A,B), while 2‐h glucose, HbA1c, LDL, TG, and total cholesterol showed no differences (Table 1). In LFD, fasting glucose was not correlated with IENFD (Figure 5C), but HDL showed a positive correlation (*r* ^2^ = 0.13, *p* < 0.05, Figure 5D). A correlation matrix showed direct associations between 1‐deoxySO, 1‐deoxySA, 1‐deoxySL, ala, ser, and fasting glucose, and inverse correlations between 1‐deoxySO, 1‐deoxySA, 1‐deoxySL, HDL, and distal IENFD (supplemental Figure S3).

### 3.6 Comorbid Arterial Hypertension and Hypercholesterolemia Are Associated With Lower Epidermal Innervation

To investigate a potential link between components of the metabolic syndrome and either IENFD or 1‐deoxySL levels, we stratified our cohort into the following subgroups: (1) patients with and without arterial hypertension and (2) patients with and without hypercholesterolemia based on medical history and previously documented diagnoses. IENFD was lower in the previously diagnosed hypertension or hypercholesterolemia group (*p* < 0.05 each, Table S2), whereas 1‐deoxySL levels did not differ between groups (Table S2). Corresponding Hodges–Lehmann median differences with 95% confidence intervals are provided in Table S2.

### 3.7 Multivariate Regression Confirms 1‐DeoxySL and Sex as Independent Predictors of IENFD

To address the limitations of group‐based comparisons and better reflect the continuous distribution of IENFD, we performed a multivariate linear regression analysis including age, sex, BMI, glucose metabolism, lipid parameters, 1‐deoxySL levels, and ala/ser ratio. The final model identified 1‐deoxySL and sex as independent predictors of IENFD. Higher 1‐deoxySL levels correlated with lower IENFD (*B* = −5.44, *p* < 0.01), and male sex was also independently correlating with reduced IENFD (*B* = −1.97, *p* < 0.05). The model explained 24% of the variance in IENFD (adjusted R^2^ = 0.22, *p* < 0.001, Table 3). No associations were observed for BMI, glucose metabolism (fasting and 2‐h glucose level in OGTT, HbA1c), lipid parameters (LDL, HDL, TG), or the ala/ser ratio (supplemental Table S3).

**TABLE 3:** Regression model for predictors of IENFD.

Model	Predictor	Adjusted *R* ^2^	*B*	*β*	*p*	95% CI
Model 1	1‐DeoxySL	0.15	−6.43	−0.41	*p* < 0.001	(−9.95;—2.92)
Model 2	1‐DeoxySL	0.22	−5.44	−0.35	*p* < 0.01	(−8.91;—1.97)
	Sex		−1.97	−0.28	*p* < 0.05	(−3.53; —0.42)

Abbreviations: 1‐deoxySL = 1‐deoxysphingolipids, CI = confidence interval, IENFD = intraepidermal nerve fiber density.

## 4 Discussion

We investigated plasma profiles of 1‐deoxy SL and AA, and serum levels of glucose and lipoproteins as potential contributors to skin denervation in patients with SFN. Our data reveal distinct correlations between these blood parameters and IENFD [28].

Compared to healthy controls reported in the literature [29, 30], we show elevated plasma 1‐deoxySL levels in patients with idiopathic SFN, highlighting the role of 1‐deoxySL in epidermal denervation also beyond the context of HSAN1 [10, 12]. These findings are consistent with previous research linking 1‐deoxySL levels to IENFD in SFN patients with distinct medical histories and reduced IENFD [8]. A subgroup analysis of SFN patients with LFD showed higher plasma 1‐deoxySL levels in the LFD group compared to the HFD group, with an inverse correlation between plasma 1‐deoxySL and IENFD in the LFD group. This suggests that the neurotoxic effects of 1‐deoxySL may contribute to the degradation of small caliber nerve fibers in SFN.

In addition to their structural effects, 1‐deoxySL correlated with altered thermal pain perception in patients with reduced IENFD. In line with previous findings [2], the observed positive correlation between elevated 1‐deoxySL levels and cold pain thresholds as well as paradoxical heat sensation in the LFD group suggest a potential association with small fiber function; however, these data do not allow for conclusions regarding causality.

1‐deoxySL are formed due to a metabolic imbalance in the ala and ser metabolism. Elevated ala and reduced ser levels promote the formation of these lipids, ultimately leading to epidermal denervation [12, 31]. Specifically in LFD patients, we observed a weak inverse correlation between 1‐deoxySL and the ala/ser ratio consistent with previous studies [9, 12], but not a direct association between blood ala/ser and epidermal denervation [9]. Given that an elevated ala/ser ratio promotes 1‐deoxySL formation, which is associated with SFN severity, a link between these parameters would be expected. Although our data support the mechanistic role of the ala/ser ratio in promoting 1‐deoxySL formation, the lack of a correlation with IENFD could be obscured by cohort‐specific characteristics and the multifactorial nature of small fiber pathology.

Interventional studies in HSAN1 have shown that lowering the ala/ser ratio by oral ser supplementation reduces 1‐deoxySL and improves clinical symptoms, suggesting that the neurotoxic effects of 1‐deoxySL may be reversible [32, 33]. However, it remains uncertain whether these findings are applicable to SFN patients and could serve as a potential therapeutic option. Based on our results, dietary modulation of ser levels may be worth exploring as a future therapeutic approach in SFN, pending validation in controlled clinical trials.

Research on DM, the leading cause of secondary SFN [34, 35], has shown that dysregulated metabolic processes promote SL accumulation [23]. Beyond DM, metabolic dysfunction is also assumed to contribute to the development of idiopathic SFN [36, 37]. Prediabetes, in particular, has been controversially discussed as a potential factor influencing skin innervation and promoting SFN development [35, 38, 39]. Earlier studies reported a low prevalence of neuropathy in prediabetes [40], while more recent evidence indicates that skin denervation and SFN symptoms may already be present in this condition [38]. In our study, patients in the LFD subgroup presented higher fasting plasma glucose levels than those in the HFD group, indicating that hyperglycemia could contribute to epidermal denervation [41]. These results support a potential pathophysiological link between small fiber pathology and metabolic dysregulation, even in the absence of manifest DM. Furthermore, IENFD was reduced in patients with arterial hypertension and hypercholesterolemia, while 1‐deoxySL levels remained unchanged. This finding indicates that small fiber pathology in these patients may be influenced by additional metabolic or vascular factors beyond those directly related to 1‐deoxySL metabolism.

Our study has several limitations. As described earlier [2], genetic analysis revealed a single SPTLC1 variant in one patient without clinical signs of HSAN1 or relevant family history, suggesting no pathogenic relevance of SPTLC1/SPTLC2 variants in this cohort. Although we identified weak correlations between plasma 1‐deoxySL levels, their synthesis parameters, epidermal denervation, and sensory profiles, our data do not allow causal conclusions regarding the role of 1‐deoxySL in SFN symptom development and maintenance. We did not include age‐ and sex‐matched healthy controls analyzed in the same batch, limiting direct comparisons. Instead, elevated 1‐deoxySL levels in patient and control comparisons were estimated from published values. This limitation, along with the inherent heterogeneity of the idiopathic SFN cohort, restricts the generalizability of our findings. In addition, subgroup analysis addressing arterial hypertension and hypercholesterolemia was based on small and unequal group sizes, which may limit the statistical power and generalizability of these findings.

Still, our data highlight the relevance of parameters such as plasma 1‐deoxySL, AA profiles, serum glucose, and lipoproteins in SFN. The alterations of these parameters observed in the LFD subgroup, along with their association with epidermal denervation, suggest a potential role for this metabolic axis in SFN pathophysiology. In patient care, our findings support incorporating metabolic screening into the diagnostic work‐up for SFN patients, even in the absence of overt DM.

## Author Contributions

All authors have written and/or edited the manuscript. Patients were recruited by N.E., C.M.z.A., L.K., C.S., and N.Ü. T.H. performed the plasma SL and AA analysis. Data analysis and interpretation were done by N.E., C.M.z.A., L.K., T.H., and N.Ü. T.H., C.S., and N.Ü. designed the study concept. T.H. and N.Ü. raised funding.

## Funding

This work was supported by Deutsche Forschungsgemeinschaft UE171‐3/1, UE171‐15/1; Schweizerischer Nationalfonds zur Förderung der Wissenschaftlichen Forschung, SNF 310030_215134; European Joint Program on Rare Diseases, EJP RD+SNF 32ER30_187505; and Interdisciplinary Center for Clinical Research, Z‐2/CSP_22.

## Conflicts of Interest

The authors declare no conflicts of interest.

## Supporting information


**Figure S1:** Ala/ser ratio and IENFD. The scatter plots show (A) the total levels of ala, (B) ser, and (C) the ala/ser ratio without intergroup difference between LFD and HFD patients. Number of samples investigated: LFD = 38, HFD = 37. ala/ser = alanine/serine, HFD = high fiber density, LFD = low fiber density.

**Figure S2:** Plasma AA in patients with LFD compared to HFD. The scatter plots (A and B) show the AA without intergroup difference between LFD and HFD patients. Number of samples investigated: LFD = 38, HFD = 37. AA = amino acids, Arg = arginine, Asp = aspartic acid, Cys = cysteine, Gly = glycine, HFD = high fiber density, His = histidine, Ile = isoleucine, Leu = leucine, LFD = low fiber density, Lys = lysine, Met = methionine, Phe = phenylalanine, Pro = proline, Thr = threonine, Tyr = tyrosine, Val = valine.

**Figure S3:** Correlation matrix of all parameters in patients with LFD. Cluster 1 shows a direct association between 1‐deoxySO, 1‐deoxySA, 1‐deoxySL, ala, ser, and fasting glucose levels. Cluster 2 shows an inverse correlation between 1‐deoxySO, 1‐deoxySA, 1‐deoxySL, HDL, and distal IENFD. Number of samples investigated: LFD = 38. 1‐deoxySA = 1‐deoxy‐sphinganine, 1‐deoxySL = 1‐deoxysphingolipids, 1‐deoxySO = 1‐deoxy‐sphingosine (14Z), ala = alanine, FPG = fasting plasma glucose, HDL = high density lipoprotein, IENFD = intraepidermal nerve fiber density, LFD = low fiber density, ser = serine.

**Table S1:** Correlation matrix of QST sensory profiles and SL.

**Table S2:** Comparison of IENFD and 1‐deoxySL levels between patients with and without arterial hypertension or hypercholesterolemia.

**Table S3:** Multivariate regression of demographic and metabolic parameters before stepwise selection.

## Data Availability

The data that support the findings of this study are available on request from the corresponding author. The data are not publicly available due to privacy or ethical restrictions.
