# Supplementary figures and images for: Blood 1‐Deoxysphingolipid Levels Are Associated With Epidermal Denervation in Small Fiber Neuropathy
Source: J Peripher Nerv Syst. 2025 Dec 11;30(4):e70089. doi: 10.1111/jns.70089 (PMC12696513; doi:10.1111/jns.70089)

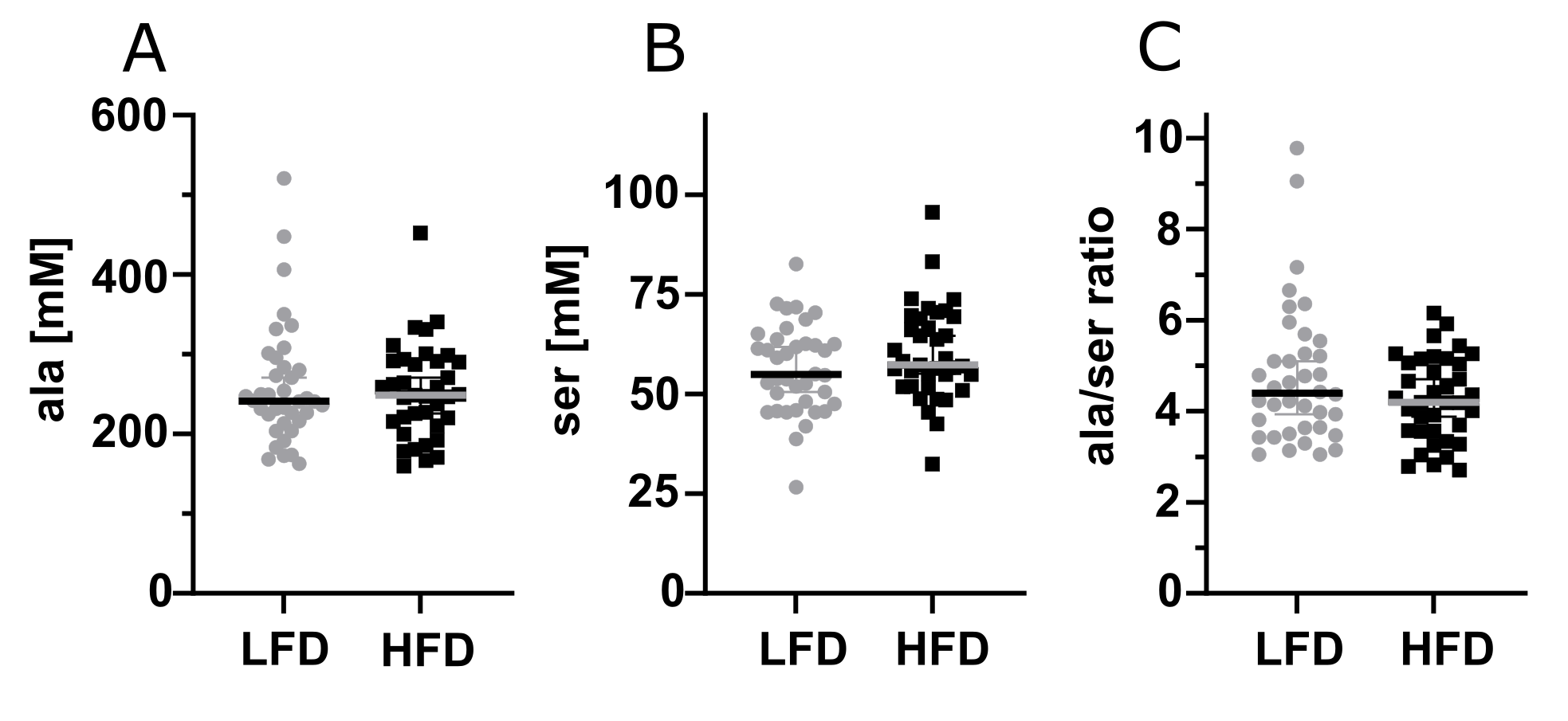

Supplement: Supplementary file 1 — Figure S1: Ala/ser ratio and IENFD. The scatter plots show (A) the total levels of ala, (B) ser, and (C) the ala/ser ratio without intergroup difference between LFD and HFD patients. Number of samples investigated: LFD = 38, HFD = 37. ala/ser = alanine/serine, HFD = high fiber density, LFD = low fiber density. [file JNS-30-0-s002.png]

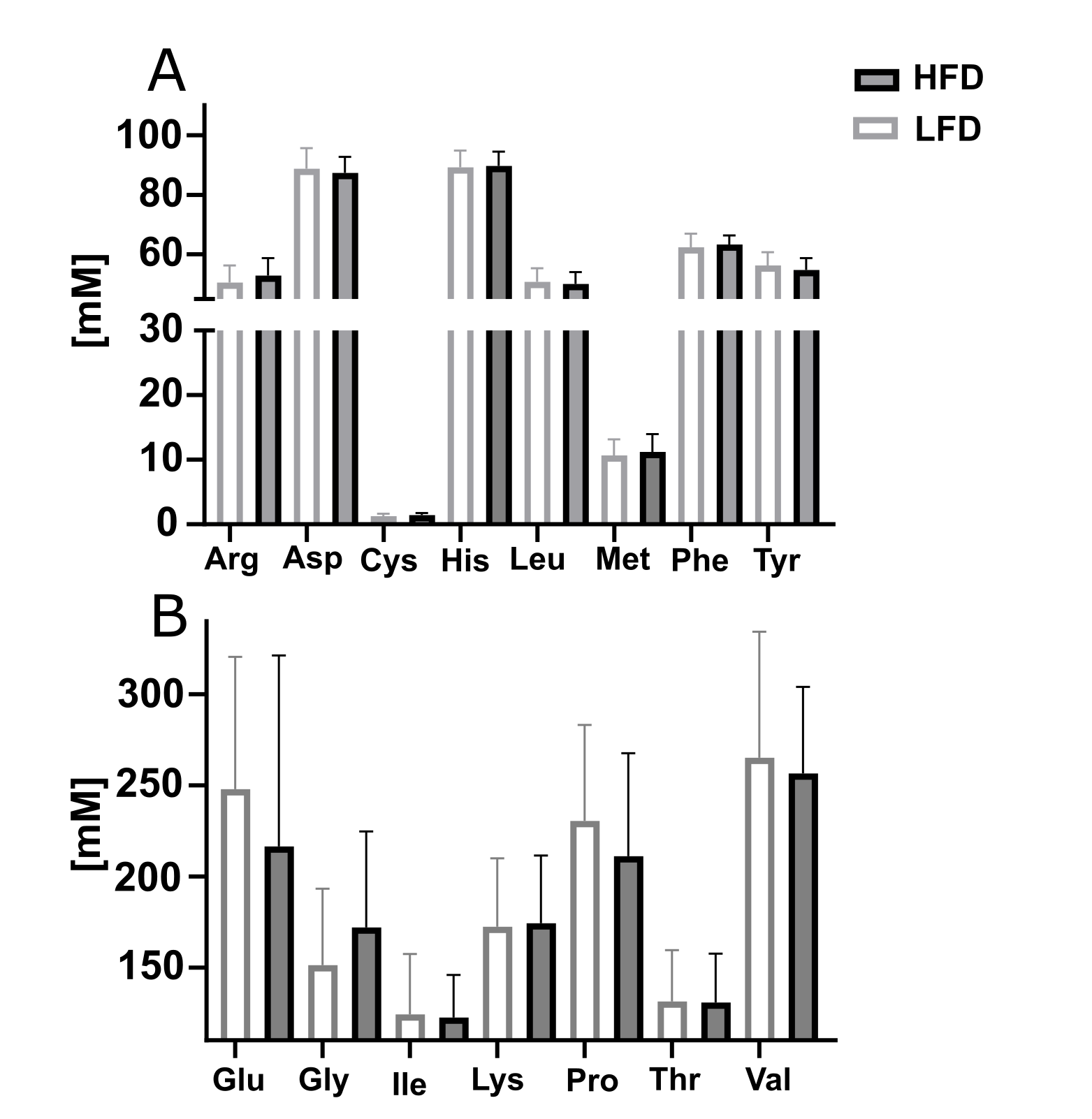

Supplement: Supplementary file 2 — Figure S2: Plasma AA in patients with LFD compared to HFD. The scatter plots (A and B) show the AA without intergroup difference between LFD and HFD patients. Number of samples investigated: LFD = 38, HFD = 37. AA = amino acids, Arg = arginine, Asp = aspartic acid, Cys = cysteine, Gly = glycine, HFD = high fiber density, His = histidine, Ile = isoleucine, Leu = leucine, LFD = low fiber density, Lys = lysine, Met = methionine, Phe = phenylalanine, Pro = proline, Thr = threonine, Tyr = tyrosine, Val = valine. [file JNS-30-0-s006.png]

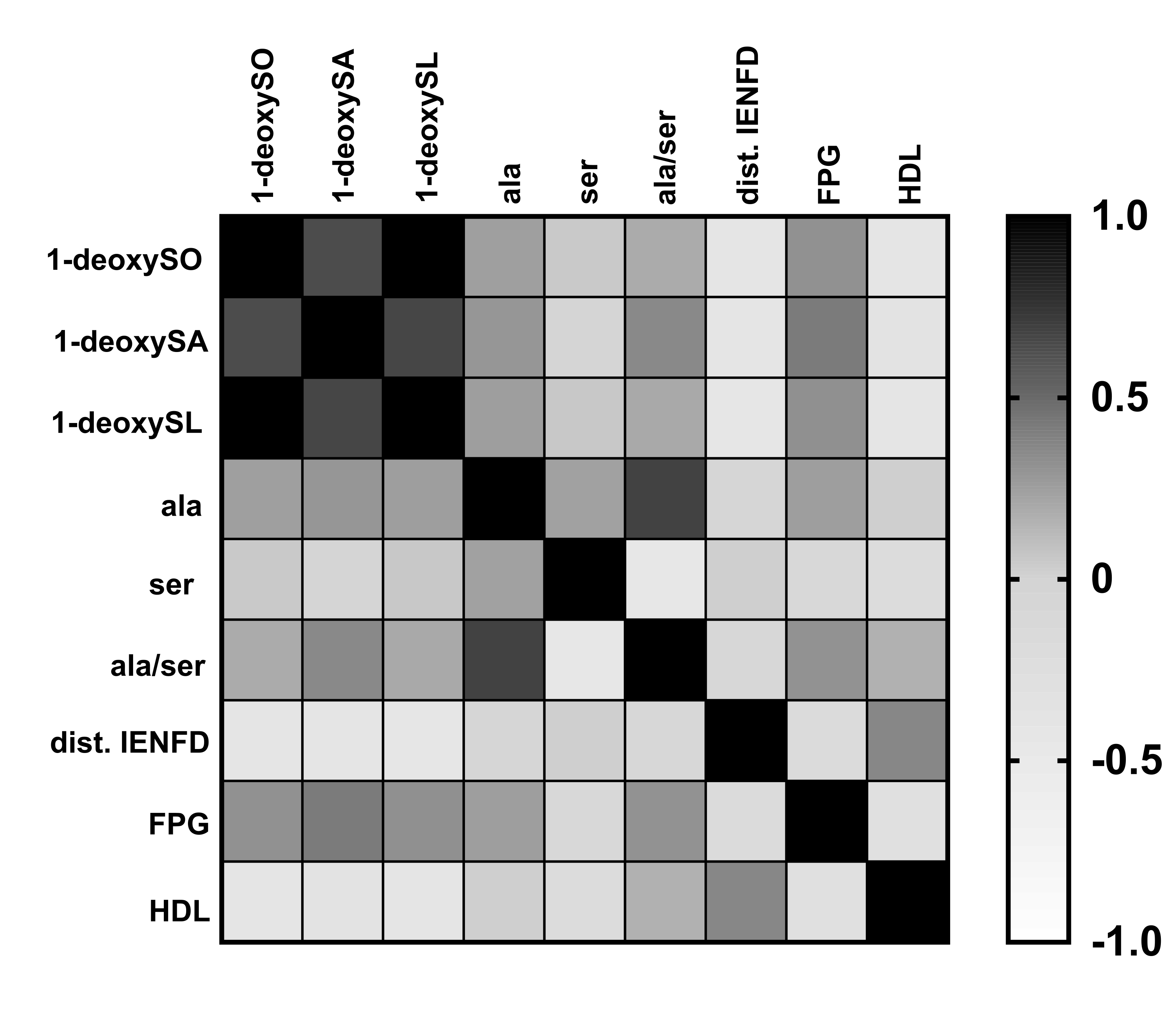

Supplement: Supplementary file 3 — Figure S3: Correlation matrix of all parameters in patients with LFD. Cluster 1 shows a direct association between 1‐deoxySO, 1‐deoxySA, 1‐deoxySL, ala, ser, and fasting glucose levels. Cluster 2 shows an inverse correlation between 1‐deoxySO, 1‐deoxySA, 1‐deoxySL, HDL, and distal IENFD. Number of samples investigated: LFD = 38. 1‐deoxySA = 1‐deoxy‐sphinganine, 1‐deoxySL = 1‐deoxysphingolipids, 1‐deoxySO = 1‐deoxy‐sphingosine (14Z), ala = alanine, FPG = fasting plasma glucose, HDL = high density lipoprotein, IENFD = intraepidermal nerve fiber density, LFD = low fiber density, ser = serine. [file JNS-30-0-s004.png]
