# Supplementary material for: Blood 1‐Deoxysphingolipid Levels Are Associated With Epidermal Denervation in Small Fiber Neuropathy
Source: J Peripher Nerv Syst. 2025 Dec 11;30(4):e70089. doi: 10.1111/jns.70089 (PMC12696513; doi:10.1111/jns.70089)
Supplement: Supplementary file 4 — Table S1: Correlation matrix of QST sensory profiles and SL. [file JNS-30-0-s005.docx]

**Supplemental Table 1** Correlation matrix of QST sensory profiles and SL.

|  | **HFD** | | | **LFD** | | |
| --- | --- | --- | --- | --- | --- | --- |
|  | 1deoxy-sphin-gosine (14Z) | 1deoxy-sphinga-nine | 1deoxy-sphingoli-pids, total | 1-deoxy-sphingosine (14Z) | 1-deoxy-sphinganine | 1-deoxy-sphingolipids, total |
| CDT | 0.31 | 0.30 | 0.31 | -0.01 | 0.03 | -0.01 |
| WDT | -0.04 | 0.03 | -0.02 | -0.07 | 0.04 | -0.06 |
| TSL | 0.13 | 0.12 | 0.14 | -0.12 | 0.10 | -0.11 |
| PHS | 0.11 | 0.12 | 0.12 | **0.45*** | **0.51*** | **0.46*** |
| CPT | -0.14 | -0.01 | -0.12 | **0.56**** | 0.36 | **0.55*** |
| HPT | 0.11 | 0.20 | 0.13 | -0.25 | -0.08 | -0.24 |
| MDT | -0.20 | -0.20 | -0.20 | -0.19 | 0.01 | -0.19 |
| MPT | -0.06 | -0.10 | -0.06 | -0.27 | -0.05 | -0.27 |
| MPS | -0.39 | -0.25 | -0.38 | -0.05 | -0.01 | -0.04 |
| VDT | 0.14 | 0.33 | 0.17 | 0.04 | -0.16 | 0.02 |
| PPT | -0.11 | -0.02 | -0.10 | 0.09 | 0.18 | 0.10 |

Using z values published in [2], we correlated all QST parameters with SL in LFD and HFD subgroup. Correlation coefficients *r* are shown in bold in case of p < 0.05. Number of samples investigated: LFD = 21, HFD = 23. Abbreviations: CDT = cold detection threshold, CPT = cold pain threshold, HFD = high fiber density, HPT = heat pain threshold, LFD = low fiber density, MDT = mechanical detection threshold, MPT = mechanical pain threshold, MPS = mechanical pain sensitivity, PHS = paradoxical heat sensation, PPT = pressure pain threshold, QST = quantitative sensory testing, SL = sphingolipids, TSL = thermal sensory limen, VDT = vibration detection threshold, WDT = warm detection threshold. * p < 0.05; ** p < 0.01.
