# Supplementary material for: Blood 1‐Deoxysphingolipid Levels Are Associated With Epidermal Denervation in Small Fiber Neuropathy
Source: J Peripher Nerv Syst. 2025 Dec 11;30(4):e70089. doi: 10.1111/jns.70089 (PMC12696513; doi:10.1111/jns.70089)
Supplement: Supplementary file 5 — Table S2: Comparison of IENFD and 1‐deoxySL levels between patients with and without arterial hypertension or hypercholesterolemia. [file JNS-30-0-s001.docx]

## Supplemental Table 2: Comparison of IENFD and 1-deoxySL levels between patients with and without arterial hypertension or hypercholesterolemia.

| **Analyte** | **Respective comorbid condition** | **Median (comorbidity present)** | **Median (comorbidity absent)** | **Hodges–Lehmann difference (95% CI)** | **p-value** |
| --- | --- | --- | --- | --- | --- |
| IENFD (fibers/mm) | Arterial hypertension | 5.40 | 2.15 | −2.00 (−4.400 to −1.000) | p < 0.05 |
|  | Hypercholesterolemia | 5.40 | 2.10 | −3.10 (−5.700 to −1.000) | p < 0.05 |
| 1-deoxySO | Arterial hypertension | 0.29 | 0.39 | +0.09 (−0.054 to 0.247) | n.s. |
|  | Hypercholesterolemia | 0.29 | 0.54 | +0.22 (−0.078 to 0.578) | n.s. |
| 1-deoxySA | Arterial hypertension | 0.04 | 0.04 | −0.00 (−0.027 to 0.016) | n.s. |
|  | Hypercholesterolemia | 0.04 | 0.06 | +0.02 (−0.018 to 0.044) | n.s. |
| 1-deoxySL | Arterial hypertension | 0.33 | 0.44 | +0.11 (−0.121 to 0.301) | n.s. |
|  | Hypercholesterolemia | 0.33 | 0.56 | +0.24 (−0.104 to 0.628) | n.s. |

Data are given as median. Exact two-tailed Mann–Whitney U tests were applied. Hodges–Lehmann median differences with 95 % confidence intervals are shown. Abbreviations: 1-deoxySO = 1deoxy-sphin-gosine (14Z), 1-deoxySA = 1deoxy-sphinga-nine, 1-deoxySL = 1-deoxysphingolipids.
