# Supplementary material for: Blood 1‐Deoxysphingolipid Levels Are Associated With Epidermal Denervation in Small Fiber Neuropathy
Source: J Peripher Nerv Syst. 2025 Dec 11;30(4):e70089. doi: 10.1111/jns.70089 (PMC12696513; doi:10.1111/jns.70089)
Supplement: Supplementary file 6 — Table S3: Multivariate regression of demographic and metabolic parameters before stepwise selection. [file JNS-30-0-s003.docx]

**Supplemental Table 3** Multivariate regression of demographic and metabolic parameters before stepwise selection.

| **Predictor** | **Adjusted R²** | **B** | **Beta** | **p values** | **95% CI** |
| --- | --- | --- | --- | --- | --- |
| Age | 0.20 | - 0.07 | - 0.22 | n.s. | [- 0.14; 0.01] |
| Sex | 0.20 | - 1.56 | - 0.22 | n.s. | [- 3.43; 0.32] |
| BMI | 0.20 | - 0.04 | - 0.04 | n.s. | [- 0.29; 0.21] |
| HDL | 0.20 | 0.79 | 0.10 | n.s. | [-1.88;  3.45] |
| LDL | 0.20 | - 0.02 | - 0.01 | n.s. | [- 1.16; 1.12] |
| TG | 0.20 | 0.58 | 0.13 | n.s. | [- 0.79; 1.95] |
| HbA1c | 0.20 | - 0.35 | - 0.04 | n.s. | [- 2.46;  1.77] |
| oGTT, fasting | 0.20 | - 0.06 | - 0.17 | n.s. | [- 0.16; 0.03] |
| oGTT, 2h | 0.20 | 0.01 | 0.08 | n.s. | [- 0.02; 0.05] |
| 1-deoxySL | 0.20 | - 6.52 | -0.41 | p < 0.05 | [- 12.03;  - 1.01] |
| Ala/ser | 0.20 | 0.48 | 0.18 | n.s. | [- 0.37; 1.32] |

Abbreviations: ala/ser = alanine/serine (ratio), BMI = body mass index, CI = confidence interval, 1-deoxySL = 1-deoxysphingolipids, HbA1c = hemoglobin A1c, HDL = high density lipoprotein, IENFD = intraepidermal nerve fiber density, LDL = low density lipoprotein, n.s. = not significant, OGTT = oral glucose tolerance test, TG = triglycerides.
